# Complexity analysis with chaos control: A discretized ratio-dependent Holling-Tanner predator-prey model with Fear effect in prey population

**DOI:** 10.1371/journal.pone.0324299

**Published:** 2025-06-05

**Authors:** Md. Mutakabbir Khan, Md. Jasim Uddin

**Affiliations:** Department of Mathematics, University of Dhaka, Dhaka, Dhaka, Bangladesh; University of Nottingham, CHINA

## Abstract

This study explores a novel two-dimensional discrete-time ratio-dependent Holling-Tanner predator-prey model, incorporating the impact of the Fear effect on the prey population. The study focuses on identifying stationary points and analyzing bifurcations around the positive fixed point, with an emphasis on their biological significance. Our examination of bifurcations at the interior fixed point uncovers a variety of generic bifurcations, including one-parameter bifurcations, period-doubling, and Neimark-Sacker bifurcations. To further understand NS bifurcation, we establish non-degeneracy condition. The system’s bifurcating and fluctuating behavior is managed using Ott—Grebogi—Yorke (OGY) control technique. From an ecological perspective, these findings underscore the substantial role of the Fear effect in shaping predator-prey dynamics. The research is extended to a networked context, where interconnected prey-predator populations demonstrate the influence of coupling strength and network structure on the system’s dynamics. The theoretical results are validated through numerical simulations, which encompass local dynamical classifications, calculations of maximum Lyapunov exponents, phase portrait analyses, and bifurcation diagrams.

## Introduction

The intricate nature of population models has long captivated researchers’ attention [1–3]. These models delve into various aspects of population dynamics, including population size, age structure, and other ecological factors. In predator-prey systems involving multiple interdependent species, external environmental influences—such as seasonal changes, predation, and time delays—can lead to chaotic behaviors or periodic oscillations [4,5]. The transition between stabilizing and destabilizing states, often driven by density-dependent mechanisms, underpins the emergence of bifurcations and chaotic dynamics in these models [6,7]. Deterministic chaos has become a fascinating and active area of exploration in ecology, mathematics, and physics, as ecological systems inherently possess feedback mechanisms conducive to chaotic phenomena [33,34,37].

In biological contexts, many models consider time as a continuous variable [5,6,8], reflecting scenarios with overlapping generational events such as continuous birth and death cycles. However, this approach falls short in situations where continuous-time assumptions are inadequate, such as the reproductive patterns of certain species like fish. Discrete-time population models address such scenarios by representing events at specific time intervals, offering a more natural framework for studying biological processes occurring in discrete phases. Discrete-time models are especially well-suited for ecological systems, as they effectively capture seasonal reproduction patterns [44,45]. Moreover, discrete-time models are known for their ability to exhibit rich dynamical behaviors—such as period-doubling bifurcations, Neimark–Sacker bifurcations, and chaotic dynamics—which are crucial for capturing the intricate interactions between species [46]. These complex patterns are often challenging to detect in continuous models, thereby making the discrete framework more advantageous for our analysis.

Liu [9] investigated the existence of periodic solutions within a discrete semi-ratio-dependent prey-predator framework. Huo and Li [10] employed Lyapunov functions to derive conditions that guarantee the global stability of solutions in a delayed discrete prey-predator system. Chen [11] proposed a discrete prey-predator model and established stability criteria for equilibrium in both periodic and non-autonomous cases. Liao *et al*. [12] concentrated on a discrete model involving one predator and two prey species, outlining conditions for the local asymptotic stability of equilibrium points. Fan and Li [13] identified sufficient conditions for permanence in a delayed discrete prey-predator system with a Holling type III functional response. However, despite these contributions, the exploration of the dynamic behaviors of discrete-time prey-predator systems—particularly with respect to bifurcations and chaotic dynamics—remains relatively underdeveloped [14–16,43].

In recent years, researchers have shown an increasing interest in Leslie-type predator-prey models as a robust framework for exploring the intricate dynamical interactions between predator and prey populations. The dynamic behavior of such systems is largely determined by the functional response of the predator, which characterizes how predator consumption rates vary with prey density. Among the various functional responses, the Holling type II response has emerged as the most widely applied, particularly in the study of arthropod predators, due to its practical relevance in ecological modeling. When this functional response is incorporated into the Leslie framework, it forms what is commonly referred to as the Holling-Tanner model.

This model serves as a cornerstone for understanding complex predator-prey interactions, capturing both stability and instability under various ecological scenarios. Its importance has attracted the attention of distinguished ecologists and mathematicians, who have extensively analyzed its behavior under different assumptions and conditions. These studies have provided significant insights into population dynamics, highlighting the Holling-Tanner model as a versatile and foundational tool in the field of mathematical ecology [17–19].

Recently, only a limited number of studies in the literature have focused on discrete-time Holling-Tanner models and their potential for exhibiting chaotic dynamics [20–22]. For example, a discrete-time predator-prey system incorporating Holling and Leslie functional responses, with constant-yield prey harvesting, was analyzed in [20]. Similarly, the study in [21] explored the dynamical properties of a discrete Holling-Tanner model, while [22] examined a discrete predator-prey model featuring a modified Holling-Tanner functional response.

These investigations primarily centered on understanding the system’s stability properties and the occurrence of bifurcations, particularly period-doubling bifurcations and Neimark-Sacker bifurcations. By employing the center manifold theory, the authors derived conditions for these bifurcations and explored their directional behavior, shedding light on the rich dynamics of such models. This emerging area of research highlights the importance of discrete-time Holling-Tanner frameworks for capturing complex predator-prey interactions and their transitions to chaos under varying ecological and harvesting conditions.

The Allee effect, a fundamental concept in mathematical biology, significantly influences population dynamics and enhances the realism of predator-prey models by modifying the traditional Lotka-Volterra system. This effect, stemming from factors such as mate selection challenges, inbreeding, reduced social cooperation, predator avoidance, and resource competition [28,29,35,36], has been documented across diverse species, including plants, insects, marine invertebrates, birds, and mammals [30]. Studies reveal its dual role in stabilizing or destabilizing population systems while introducing complex and often unpredictable dynamics [31,32]. The fear effect, which highlights the non-lethal influence of predators on prey populations, has become a focal point of research in mathematical ecology. It describes how the presence or perceived threat of predators can alter prey behavior, physiology, or spatial distribution, even in the absence of direct predation, shedding light on its critical role in ecological dynamics. The study [38] explores the impact of anti-predator behavior driven by the fear of predators within a Holling-Type II predator-prey model that incorporates the concept of a prey refuge. The role of fear on the growth function of prey population in a predator-prey interaction model is discussed in [39].

Previous studies on discrete predator-prey systems have largely concentrated on single systems, with limited exploration of coupled networks. However, real-world predator-prey interactions are seldom isolated and typically occur within interconnected systems, making coupled networks a more realistic depiction of ecological dynamics. Such networks capture the complexities of natural ecosystems and give rise to more intricate and diverse dynamic behaviors. Investigating coupled networks can uncover novel phenomena in predator-prey interactions, emphasizing the need for further research. Network theory offers a powerful framework for modeling and analyzing the intricate relationships and interactions within complex ecological systems [39,40]. Incorporating network structures into predator-prey models moves beyond traditional mean-field approximations, enabling the study of how spatial arrangements, habitat fragmentation, and the topology of interaction networks impact population dynamics, system stability, and ecosystem resilience [41,42].

In this study, inspired by the Holling-Tanner predator-prey framework with Fear effect [23,24], we introduce a novel discrete-time predator-prey model where the predator exhibits partial dependence on the prey population [25]. The model’s dynamics are thoroughly analyzed, focusing on stability and bifurcation behaviors, employing the center manifold theorem and bifurcation theory. Examining the ecological consequences of these dynamics provides critical insights into predator-prey relationships and informs potential management strategies. Additionally, we explore the application of the OGY method for chaos control.

The structure of this paper is organized as follows: Sect 1 provides a brief overview of the proposed model. In Sect 2, the fixed points of the discrete system are identified, and their corresponding stability conditions are analyzed. Sect 3 establishes the precise criteria for the onset of period-doubling and Neimark-Sacker bifurcations. In Sect 4, we conduct an in-depth analysis of complex networks within the context of a coupled dynamical network based on the discrete predator-prey system. Numerical experiments, presented in Sect 5, are used to validate the theoretical findings, highlighting phenomena such as period-doubling bifurcations, Neimark-Sacker bifurcations, and chaotic dynamics. Strategies for chaos control are explored in Sect 6. Finally, Sect 7 presents concluding remarks and summarizes the key outcomes of the study.

## 1 Formulation of model

The structure of the ratio-dependent Holling-Tanner predator-prey model with Fear effect is expressed as:

$$\left\{ \begin{array}{c} \frac{dx}{dt}=rx\left(1-\frac{x}{k}\right)\frac{1}{1+Ay}-\frac{pxy}{x+By},\\ \frac{dy}{dt}=ay\left(1-\frac{\beta y}{x}\right). \end{array}\right.$$ (1)

The initial populations are given as x(0)>0 and y(0)>0, where *x* and *y* represent the population densities of the prey and predator species, respectively, at any time *t*. The parameters *r* and *a* denote the intrinsic growth rates of the prey and predator populations, respectively, while *k* represents the carrying capacity of the prey’s environment. The predator’s consumption of prey follows a ratio-dependent Holling type II functional response, given by $\frac{px}{x+By}$. Here, *p* denotes the maximum per capita consumption rate of predators, while *B* represents the prey density required to reach half of this maximum consumption rate. Additionally, the term $\frac{1}{1+Ay}$ incorporates the Fear effect on the prey population, where *A*>0 is the constant representing the strength of the Fear effect. The units of r, p, β and *a* are time^−1^. Also, the parameters *k* and *B*, including the prey and predator populations, have the same unit, which is the number of individuals.

In (1), we apply the forward Euler method with a step size *h* to derive the following discrete predator-prey system:

$$\begin{array}{c} x_{n+1}=x_{n}+h\left[rx_{n}\left(1-\frac{x_{n}}{k}\right)\left(\frac{1}{1+Ay_{n}}\right)-\frac{px_{n}y_{n}}{x_{n}+By_{n}}\right],\\ y_{n+1}=y_{n}+h\left[ay_{n}\left(1-\frac{\beta y_{n}}{x_{n}}\right)\right]. \end{array}$$ (2)

## 2 Fixed points and their stability analysis

Fixed points, also known as equilibrium points or steady states, are essential to understand how systems evolve over time. At these points, the system remains unchanged because the equations governing it show no movement or variation. They offer valuable insights into a system’s long-term behavior, helping us understand its stability and predict how it will act under different conditions. Fixed points are used in fields like physics, biology, engineering, and economics to design controls, study stability, and anticipate how systems will respond in the real world.

### 2.1 Existence of fixed points

The fixed points of model (2) are found by solving the following set of equations:

$$\left\{ \begin{array}{c} x^{*}=x^{*}+h\left[rx^{*}\left(1-\frac{x^{*}}{k}\right)\left(\frac{1}{1+Ay^{*}}\right)-\frac{px^{*}y^{*}}{x^{*}+By^{*}}\right],\\ y^{*}=y^{*}+h\left[ay^{*}\left(1-\frac{\beta y^{*}}{x^{*}}\right)\right]. \end{array}\right.$$ (3)

The fixed points of model (2) are obtained through direct computation as follows: The system exhibits the following fixed points:

1. Semi-trivial fixed point: $\chi_{0}=\left(k,0\right)$, representing the absence of predators.
2. Coexistence fixed point: $\chi_{1}=\left(\beta \cdot y^{*},y^{*}\right)$, where
  $$y^{*}=\frac{k(r(\beta +B)-p)}{Akp+\beta r(\beta +B)}.$$

Here, $\chi_{1}$ represents the coexistence states where both the prey and predator populations sustain themselves. The coexistence equilibrium $\chi_{1}$ is achievable since all parameter values are positive and the only restriction is r(β+B)>p.

### 2.2 Evaluating stability for fixed points

To investigate the stability of the system (2) at the equilibrium point χ(x,y), we conduct an eigenvalue analysis of the Jacobian matrix evaluated at this point. The local stability of χ(x,y) is fundamentally governed by the eigenvalues’ magnitudes. The Jacobian matrix at the equilibrium is given by:

$$J(x,y)=\left(\begin{array}{cc} \frac{hr(k-2x)}{Aky+k}-\frac{Bhpy^{2}}{(By+x{)}^{2}}+1 & hx\left(-\frac{Ar(k-x)}{k(Ay+1{)}^{2}}-\frac{px}{(By+x{)}^{2}}\right)\\ \frac{a\beta hy^{2}}{x^{2}} & \frac{ah(x-2\beta y)}{x}+1 \end{array}\right).$$ (4)

The characteristic polynomial of the Jacobian matrix corresponding to (4) is given by

$$\begin{array}{cc} P(\rho ) & =\rho^{2}-\Lambda \rho +\Upsilon =0, \end{array}$$ (5)

where Λ represents the trace and Υ denotes the determinant of J(x,y).

**Proposition 1.** *The fixed point $\chi_{0}$ is classified as follows: (i) saddle point under the condition $0<h<\frac{2}{r}$, (ii) source under the condition $h>\frac{2}{r}$, (iii) a non-hyperbolic point $h=\frac{2}{r}$.*

**Proof** It can be obtained that,


$$J(\chi_{0})=\left(\begin{array}{cc} 1-hr & -hp\\ 0 & ah+1 \end{array}\right).$$

The eigenvalues are $\rho_{1}=1-hr$ and $\rho_{2}=ah+1$. Clearly, $\rho_{2}>1$ and


$$|1-hr|\left\{ \begin{array}{cc} <1 & \text{if }0<h<\frac{2}{r},\\ =1 & \text{if }h=\frac{2}{r},\\ >1 & \text{if }h>\frac{2}{r}. \end{array}\right.$$

$$\begin{array}{cccc} Condition & \left|\rho_{1}|=|1-hr\right| & \left|\rho_{2}|=|ah+1\right| & TypeofFixedPoint\\ |1-hr|>1\text{ and }|ah+1|>1 & \text{Unstable } & \text{Unstable } & \text{Source}\\ |1-hr|<1\text{ and }|ah+1|>1 & \text{Stable } & \text{Unstable } & \text{Saddle}\\ |1-hr|=1\text{ or }|ah+1|=1 & \text{Non-hyperbolic } & \text{Non-hyperbolic } & \text{Non-hyperbolic} \end{array}$$

◻

Naturally, one of the eigenvalues of $J\left(\chi_{0}\right)$ is −1, while the other is not equal to ±1 when $h=\frac{2}{r}$. Consequently, a period-doubling (PD) bifurcation may occur if the parameters vary within a constrained region around ${\hat{FB}}_{\chi_{0}}$.


$${\hat{FB}}_{\chi_{0}}=\left\{(r,k,h,A,p,B,a,\beta )\in (0,+\infty ):h=\frac{2}{r}\right\}.$$

**Proposition 2.** *The following topological classification applies to the coexistence fixed point $\chi_{1}(x^{*},y^{*})$:*

*(i) source if*

*(i.a) $\varsigma^{2}-4\vartheta \geq 0$ and $h_{e}>\frac{-\varsigma +\sqrt{\varsigma^{2}-4\vartheta }}{\vartheta }$*

*(i.b) $\varsigma^{2}-4\vartheta <0$ and $h_{e}>\frac{-\varsigma }{\vartheta }$*

*(ii) sink if*

*(ii.a) $\varsigma^{2}-4\vartheta \geq 0$ and $h_{e}<\frac{-\varsigma -\sqrt{\varsigma^{2}-4\vartheta }}{\vartheta }$*

*(ii.b) $\varsigma^{2}-4\vartheta <0$ and $h_{e}<\frac{-\varsigma }{\vartheta }$*

*(iii) non-hyperbolic if*

*(iii.a) $\varsigma^{2}-4\vartheta \geq 0$ and $h_{e}=\frac{-\varsigma \pm \sqrt{\varsigma^{2}-4\vartheta }}{\vartheta };h_{e}\neq \frac{-2}{\varsigma },\frac{-4}{\varsigma }$*

*(iii.b) $\varsigma^{2}-4\vartheta <0$ and $h_{e}=\frac{-4}{\varsigma }$.*

*(iv) saddle if otherwise*

**Proof** At $\chi_{1}(x^{*},y^{*})$, the characterizing equation looks like this:

$$F_{e}(\rho ):=\rho^{2}-(2+\varsigma h_{e})\rho +(1+\varsigma h_{e}+\vartheta {h_{e}}^{2})=0,$$ (6)

where


$$\varsigma =-\frac{2a\beta y^{*}}{x^{*}}+a+\frac{r(k-2x^{*})}{Aky^{*}+k}-\frac{Bpy^{*2}}{(By^{*}+x^{*}{)}^{2}},\;\vartheta =\frac{a\left(\frac{py^{*2}(Ay^{*}+1{)}^{2}(-Bx^{*}+2\beta By^{*}+\beta x^{*})}{x^{*}(By^{*}+x^{*}{)}^{2}}-\frac{2rx^{*}(Ay^{*}+1)}{k}+\frac{\beta ry^{*}(3Ay^{*}+4)}{k}-\frac{\beta ry^{*}(Ay^{*}+2)}{x^{*}}+Ary^{*}+r\right)}{(Ay^{*}+1{)}^{2}},\;h_{e}=h.\;$$

So $F_{e}(1)=\vartheta {h_{e}}^{2}>0$ and $F_{e}(-1)=4+2\varsigma h_{e}+\vartheta {h_{e}}^{2}$. We conclude further indicating different criteria of the stability of $\chi_{1}$. ◻

## 3 Study of bifurcations

This section delves into research examining Neimark-Sacker (NS) and period-doubling (PD) bifurcations at the fixed point $\chi_{1}(x^{*},y^{*})$ of the model, with the parameter *h* serving as a tool to trace the progression of these bifurcations.

### 3.1 Neimark-Sacker bifurcation

Here, we turn our attention to the Neimark-Sacker (NS) bifurcation, employing the parameter *h* as the bifurcation parameter. In particular, we investigate:


$${\hat{\Lambda }}_{\chi_{1}}=\left\{(r,k,h,A,p,B,a,\beta ):h=\frac{-\varsigma }{\vartheta }=h_{NS},\;\varsigma^{2}-4\vartheta <0\right\}.$$

By introducing a small perturbation, denoted as $h^{*}$, to the bifurcation parameter *h*, the model (2) can be modified and expressed as:

$$x_{n+1}=x_{n}+(h+h^{*})\left(rx_{n}\left(1-\frac{x_{n}}{k}\right)\left(\frac{1}{1+Ay_{n}}\right)-\frac{px_{n}y_{n}}{x_{n}+By_{n}}\right)\equiv f(x_{n},y_{n},h^{*}),$$ (7)

$$y_{n+1}=y_{n}+(h+h^{*})\left(ay_{n}\left(1-\frac{\beta y_{n}}{x_{n}}\right)\right)\equiv g(x_{n},y_{n},h^{*}).$$

Let $u_{n}=x_{n}\;-\;x^{*}$ and $v_{n}=y_{n}\;-\;y^{*}$, where $\left(x^{*},y^{*}\right)$ is the equilibrium point, $\chi_{1}$. Under this transformation, the equilibrium point is shifted to the origin, so $\left(u_{n},v_{n})=(0,0\right)$. Expanding the functions *f* and *g* using a third-order Taylor series, the reformulated version of the system in the model (7) is obtained.

$$u_{n+1}=\gamma_{x1}u_{n}+\gamma_{x2}v_{n}+\gamma_{x11}u_{n}^{2}+\gamma_{x12}u_{n}v_{n}+\gamma_{x22}v_{n}^{2}+\gamma_{x111}u_{n}^{3}+\gamma_{x112}u_{n}^{2}v_{n}\;\;+\gamma_{x122}u_{n}v_{n}^{2}+\gamma_{x222}v_{n}^{3}+O((\left|u_{n}\right|+\left|v_{n}\right|{)}^{4}),\;v_{n+1}=\eta_{y1}u_{n}+\eta_{y2}v_{n}+\eta_{y11}u_{n}^{2}+\eta_{y12}u_{n}v_{n}+\eta_{y22}v_{n}^{2}+\eta_{y111}u_{n}^{3}+\eta_{y112}u_{n}^{2}v_{n}\;\;+\eta_{y122}u_{n}v_{n}^{2}+\eta_{y222}v_{n}^{3}+O((\left|u_{n}\right|+\left|v_{n}\right|{)}^{4}),$$ (8)

where, all the coefficient values of Eq (8) are displayed in Table 1.

**Table 1:** Coefficient values for Eq (8), where $\varrho =\frac{k(r(\beta +B)-p)}{Akp+\beta r(\beta +B)}$ and ϖ=βϱ.

Term	Expression	Term	Expression
$\gamma_{x1}$	$\frac{hr(k-2\varpi )}{Ak\varrho +k}-\frac{Bhp\varrho^{2}}{(B\varrho +\varpi {)}^{2}}+1$	$\gamma_{x2}$	$h\varpi \left(-\frac{Ar(k-\varpi )}{k(A\varrho +1{)}^{2}}-\frac{p\varpi }{(B\varrho +\varpi {)}^{2}}\right)$
$\gamma_{x11}$	$h\left(\frac{2Bp\varrho^{2}}{(B\varrho +\varpi {)}^{3}}-\frac{2r}{Ak\varrho +k}\right)$	$\gamma_{x12}$	$\frac{h}{k(A\varrho +1{)}^{2}(B\varrho +\varpi {)}^{3}}(2A^{2}Bkp\varpi \varrho^{3}+Ak(4Bp\varpi \varrho^{2}$
			$+r(B\varrho +\varpi {)}^{3})-2Ar\varpi (B\varrho +\varpi {)}^{3}+2Bkp\varpi \varrho )$
$\gamma_{x22}$	$2x2009;h\varpi \left(\frac{A^{2}r(k-\varpi )}{k(A\varrho +1{)}^{3}}+\frac{Bp\varpi }{(B\varrho +\varpi {)}^{3}}\right)$	$\gamma_{x111}$	$-\frac{6Bhp\varrho^{2}}{(B\varrho +\varpi {)}^{4}}$
$\gamma_{x112}$	$h\left(\frac{2Ar}{k(A\varrho +1{)}^{2}}-\frac{2Bp\varrho (B\varrho -2\varpi )}{(B\varrho +\varpi {)}^{4}}\right)$	$\gamma_{x122}$	$2x2009;h\left(\frac{A^{2}r(k-2\varpi )}{k(A\varrho +1{)}^{3}}+\frac{Bp\varpi (2B\varrho -\varpi )}{(B\varrho +\varpi {)}^{4}}\right)$
$\gamma_{x222}$	$6x2009;h\varpi \left(-\frac{A^{3}r(k-\varpi )}{k(A\varrho +1{)}^{4}}-\frac{B^{2}p\varpi }{(B\varrho +\varpi {)}^{4}}\right)$		
$\eta_{y1}$	$\frac{a\beta h\varrho^{2}}{\varpi^{2}}$	$\eta_{y2}$	$a\left(h-\frac{2\beta h\varrho }{\varpi }\right)+1$
$\eta_{y11}$	$-\frac{2a\beta h\varrho^{2}}{\varpi^{3}}$	$\eta_{y12}$	$\frac{2a\beta h\varrho }{\varpi^{2}}$
$\eta_{y22}$	$-\frac{2a\beta h}{\varpi }$	$\eta_{y111}$	$\frac{6a\beta h\varrho^{2}}{\varpi^{4}}$
$\eta_{y112}$	$-\frac{4a\beta h\varrho }{\varpi^{3}}$	$\eta_{y122}$	$\frac{2a\beta h}{\varpi^{2}}$
$\eta_{y222}$	0		

The characteristic equation of the model (8) is given by $\rho^{2}-\Lambda (h^{*})\rho +\Upsilon (h^{*})=0$, where $\Lambda (h^{*})=(2+\varsigma h_{e})$ and $\Upsilon (h^{*})=(1+\varsigma h_{e}+\vartheta {h_{e}}^{2})$. The roots of the characteristic equation are expressed as


$$\rho_{1,2}(h^{*})=\frac{-\Lambda (h^{*})\pm i\sqrt{4\Upsilon (h^{*})-(\Lambda (h^{*}){)}^{2}}}{2}.$$

For $\left|\rho_{1,2}(h^{*})\right|=1$ and when h^*^ = 0, we find that $\left|\rho_{1,2}(h^{*})\right|=\sqrt{\Upsilon (h^{*})}$. Additionally, the derivative

$$l={\left[\frac{d\left|\rho_{1,2}(h^{*})\right|}{dh^{*}}\right]}_{h^{*}=0}\neq 0.$$ (9)

It is also essential to ensure that, when h^*^ = 0, the roots $\rho_{1,2}^{i}\neq 1$ for i=1,2,3,4, which implies that Λ(0)≠±2,0,1.

To investigate the normal form, let ϕ=Im(ρ1,2) and φ=Re(ρ1,2). We introduce the transformation matrix


$$T=[\begin{array}{cc} 0 & 1\\ \phi & \varphi \end{array}],$$

and apply the coordinate change


$$[\begin{array}{c} u_{n}\\ v_{n} \end{array}]=T[\begin{array}{c} {\overset{―}{x}}_{n}\\ {\overset{―}{y}}_{n} \end{array}].$$

Under this transformation, the model (8) becomes:


$$overlinex_{n+1}=\varphi {\overset{―}{x}}_{n}-\phi {\overset{―}{y}}_{n}+f_{x11}({\overset{―}{x}}_{n},{\overset{―}{y}}_{n}),$$

$${\overset{―}{y}}_{n+1}=\phi {\overset{―}{x}}_{n}+\varphi {\overset{―}{y}}_{n}+g_{y11}({\overset{―}{x}}_{n},{\overset{―}{y}}_{n}),$$

where $f_{x11}({\overset{―}{x}}_{n},{\overset{―}{y}}_{n})$ and $g_{y11}({\overset{―}{x}}_{n},{\overset{―}{y}}_{n})$ represent higher-order terms, with both variables $\left({\overset{―}{x}}_{n},{\overset{―}{y}}_{n}\right)$ contributing to these functions with a degree of at least two.

For the discriminating parameter Ω to proceed through the Neimark-Sacker bifurcation (NSB), it is imperative that Ω≠0.

$\Omega =-Re\left[\frac{(1-2\overset{―}{\rho }){\overset{―}{\rho }}^{2}}{1-\rho }\kappa_{11}\kappa_{20}\right]-\frac{1}{2}{\left|\kappa_{11}\right|}^{2}-{\left|\kappa_{02}\right|}^{2}+Re(\overset{―}{\rho }\kappa_{21}),$ where


$$\kappa_{20}=\frac{\varphi }{8}\left(2\eta_{y22}-\varphi \gamma_{x22}-\gamma_{x12}+4\phi \gamma_{x22}+i\left(4\phi \gamma_{x22}-2\gamma_{x22}-2\varphi \gamma_{x22}\right)\right)+\frac{\phi }{4}\gamma_{x12}\;\;+i\frac{1}{8}\left(4\phi \eta_{y22}+2\phi^{2}\gamma_{x22}-2\gamma_{x11}\right)+\frac{\eta_{y12}}{8}\;\;+\frac{\varphi \gamma_{x11}-2\eta_{y11}+\varphi^{3}\gamma_{x22}-\varphi^{2}\eta_{y22}-\varphi^{2}\gamma_{x12}+\varphi \eta_{y12}}{4\phi },\;\kappa_{11}=\frac{\phi }{2}(\eta_{y22}-\varphi \gamma_{x22})+i\frac{1}{2}(\phi^{2}\gamma_{x22}+\gamma_{x11}+\varphi \gamma_{x12}+\varphi^{2}\gamma_{x22})\;\;+\frac{\eta_{y11}-\varphi \gamma_{x11}+\varphi \eta_{y12}-\varphi^{2}\gamma_{x12}-2\varphi^{2}\eta_{y22}+2\varphi^{3}\gamma_{x22}}{2\phi },\;\kappa_{02}=\frac{1}{4}\phi (2\varphi \gamma_{x22}+\gamma_{x12}+\eta_{y22})+i\frac{1}{4}(\eta_{y12}+2\varphi \eta_{y22}-2\varphi \gamma_{x12}-\gamma_{x11})\;\;-\frac{\eta_{y11}-\varphi \gamma_{x11}+\varphi \eta_{y12}-\varphi^{2}\gamma_{x12}+\varphi^{2}\eta_{y22}-\varphi^{3}\gamma_{x22}}{4\phi }+\frac{1}{4}\gamma_{x22}i(\phi^{2}-3\varphi^{2}),\;\kappa_{21}=\frac{3}{8}\eta_{y222}(\phi^{2}+\varphi^{2})+\frac{\eta_{y112}}{8}+\frac{\varphi }{4}\gamma_{x112}+\frac{\varphi }{4}\eta_{y}122+\gamma_{x122}(\frac{\phi^{2}}{8}+\frac{3\varphi^{2}}{8}-\frac{\varphi }{4})\;\;+\frac{3}{8}\gamma_{x111}+i\frac{3}{8}\gamma_{x222}(\phi^{2}+2\varphi^{2})+i\frac{3\phi \varphi }{8}\gamma_{x122}-\frac{1}{8}\eta_{y122}\phi i-i\frac{3\phi \varphi }{8}\eta_{y222}-i\frac{3\eta_{y111}-3\varphi \gamma_{x111}}{8\phi }\;\;-i\frac{3\varphi \eta_{y112}-3\varphi^{2}\gamma_{x112}}{8\phi }-i\frac{3\varphi^{2}\eta_{y122}-3\varphi^{3}\gamma_{x122}}{8\phi }-i\frac{3\varphi^{3}\eta_{y222}-3\varphi^{4}\gamma_{x222}}{8\phi }.$$

Based on the preceding analysis, we state the following conclusion:

**Theorem 1.** *If Ω≠0, the system undergoes a Neimark-Sacker (NS) bifurcation at the equilibrium point $\chi_{1}(x^{*},y^{*})$ when the parameter *h* reaches the critical value ${\hat{\Lambda }}_{\chi_{1}}$. For Ω<0, the bifurcation may be subcritical, leading to the formation of a smooth closed invariant curve around the positive fixed point $\chi_{1}(x^{*},y^{*})$. On the other hand, for Ω>0, the bifurcation is likely supercritical, similarly resulting in such a closed invariant curve.*

### 3.2 Period-doubling bifurcation

In this section, we analyze the PD bifurcation by utilizing *h* as the bifurcation parameter. Specifically, we consider:


$${\hat{\Theta }}_{\chi_{1}}=\left\{(r,k,h,A,p,B,a,\beta ):h=\frac{-\varsigma \pm \sqrt{\varsigma^{2}-4\vartheta }}{\vartheta }=h_{\pm };\;\varsigma^{2}-4\vartheta \geq 0,h\neq \frac{-2}{\varsigma },\frac{-4}{\varsigma }\right\}.$$

By introducing a small perturbation, $h^{*}$, to the bifurcation parameter *h*, the system described by model (2) can be rewritten as:

$$x_{n+1}=x_{n}+(h+h^{*})\left(rx_{n}\left(1-\frac{x_{n}}{k}\right)\left(\frac{1}{1+Ay_{n}}\right)-\frac{px_{n}y_{n}}{x_{n}+By_{n}}\right)\equiv f(x_{n},y_{n},h^{*}),$$ (10)

$$y_{n+1}=y_{n}+(h+h^{*})\left(ay_{n}\left(1-\frac{\beta y_{n}}{x_{n}}\right)\right)\equiv g(x_{n},y_{n},h^{*}).$$

Now, let $u_{n}=x_{n}\;-\;x^{*}$ and $v_{n}=y_{n}\;-\;y^{*}$, where the equilibrium point is identified as $\chi_{1}(x^{*},y^{*})$. By shifting the origin to $\left(u_{n},v_{n})=(0,0\right)$, the functions *f* and *g* are expanded as a third-order Taylor series around the origin, resulting in a transformed version of the system in model (10).

$$u_{n+1}=\gamma_{x1}u_{n}+\gamma_{x2}v_{n}+\gamma_{x11}u_{n}^{2}+\gamma_{x12}u_{n}v_{n}+\gamma_{x13}u_{n}h^{*}+\gamma_{x23}v_{n}h^{*}+\gamma_{x111}u_{n}^{3}+\;\;\gamma_{x112}u_{n}^{2}v_{n}+\gamma_{x113}u_{n}^{2}h^{*}+\gamma_{x123}u_{n}v_{n}h^{*}+O((\left|u_{n}\right|+\left|v_{n}\right|+\left|h^{*}\right|{)}^{4}),\;v_{n+1}=\eta_{y1}u_{n}+\eta_{y2}v_{n}+\eta_{y11}u_{n}^{2}+\eta_{y12}u_{n}v_{n}+\eta_{y22}v_{n}^{2}+\eta_{y13}u_{n}h^{*}+\eta_{y23}v_{n}h^{*}+\eta_{y111}u_{n}^{3}\;\;+\eta_{y112}u_{n}^{2}v_{n}+\eta_{y113}u_{n}^{2}h^{*}+\eta_{y123}u_{n}v_{n}h^{*}+\eta_{y223}v_{n}^{2}h^{*}+O((\left|u_{n}\right|+\left|v_{n}\right|+\left|h^{*}\right|{)}^{4}),$$ (11)

where, all the coefficient values of Eq (11) are shown in Table 2.

**Table 2:** Coefficient expressions for Eq (11), where $\varrho =\frac{k(r(\beta +B)-p)}{Akp+\beta r(\beta +B)}$ and ϖ=βϱ.

Term	Expression
$\gamma_{x13}$	$\frac{r(k-2\varpi )}{Ak\varrho +k}-\frac{Bp\varrho^{2}}{(B\varrho +\varpi {)}^{2}}$
$\gamma_{x23}$	$\varpi \left(-\frac{Ar(k-\varpi )}{k(A\varrho +1{)}^{2}}-\frac{p\varpi }{(B\varrho +\varpi {)}^{2}}\right)$
$\gamma_{x113}$	$\frac{2Bp\varrho^{2}}{(B\varrho +\varpi {)}^{3}}-\frac{2r}{Ak\varrho +k}$
$\gamma_{x123}$	$-\frac{1}{k(A\varrho +1{)}^{2}(B\varrho +\varpi {)}^{3}}[2A^{2}Bkp\varpi \varrho^{3}+Ak(4Bp\varpi \varrho^{2}+r(B\varrho +\varpi {)}^{3})$
$-2Ar\varpi (B\varrho +\varpi {)}^{3}+2Bkp\varpi \varrho ]$	
$\eta_{y13}$	$\frac{a\beta \varrho^{2}}{\varpi^{2}}$
$\eta_{y23}$	$a-\frac{2a\beta \varrho }{\varpi }$
$\eta_{y113}$	$-\frac{2a\beta \varrho^{2}}{\varpi^{3}}$
$\eta_{y123}$	$\frac{2a\beta \varrho }{\varpi^{2}}$
$\eta_{y223}$	$-\frac{2a\beta }{\varpi }$

Let $T=[\begin{array}{cc} \gamma_{x2} & \gamma_{x2}\\ -1-\gamma_{x1} & \rho_{2}-\gamma_{x1} \end{array}]$ represent an invertible matrix. By employing the transformation


$$[\begin{array}{c} u_{n}\\ v_{n} \end{array}]=T[\begin{array}{c} {\overset{―}{x}}_{n}\\ {\overset{―}{y}}_{n} \end{array}],$$

the original model (11) is reformulated into the system:

$${\overset{―}{x}}_{n+1}=-{\overset{―}{x}}_{n}+f_{x11}(u_{n},v_{n},h^{*}),$$ (12)

$${\overset{―}{y}}_{n+1}=\rho_{2}{\overset{―}{y}}_{n}+g_{y11}(u_{n},v_{n},h^{*}).$$

Here, the new variables $\left({\overset{―}{x}}_{n},{\overset{―}{y}}_{n}\right)$ are connected to the functions *f*_*x*11_ and *g*_*y*11_, which encapsulate the terms of the transformed system (12). The pair $\left({\overset{―}{x}}_{n},{\overset{―}{y}}_{n}\right)$ is defined with a rank of at least two.

The system (12) admits a center manifold *W*^*c*^(0,0,0) in the vicinity of the equilibrium point (0,0), assuming that the parameter $h^{*}$ is near zero. This result is grounded in the application of the center manifold theorem, which simplifies the dynamics. Consequently, the system can be effectively described by:


$$W^{c}(0,0,0)=\left\{({\overset{―}{x}}_{n},{\overset{―}{y}}_{n},h^{*})\in {\mathbb{R}}^{3}:{\overset{―}{y}}_{n+1}={\overset{―}{\gamma }}_{x1}{\overset{―}{x}}_{n}^{2}+{\overset{―}{\gamma }}_{x2}{\overset{―}{x}}_{n}h^{*}+O((|{\overset{―}{x}}_{n}|+|h^{*}|{)}^{3})\right\}.$$

This formulation highlights that the dynamics of the system near the equilibrium can be captured by the reduced expressions on the center manifold.

The coefficients ${\overset{―}{\gamma }}_{x1}$ and ${\overset{―}{\gamma }}_{x2}$ are expressed as follows:


$${\overset{―}{\gamma }}_{x1}=\frac{\gamma_{x2}[(1+\gamma_{x1})\gamma_{x11}+\gamma_{x2}\gamma_{x11}]}{1-\rho_{2}^{2}}+\frac{\eta_{y22}(1+\gamma_{x1}{)}^{2}}{1-\rho_{2}^{2}}-\frac{\left(1+\gamma_{x1})[\gamma_{x12}(1+\gamma_{x1})+\gamma_{x2}\eta_{y12}\right]}{1-\rho_{2}^{2}},$$

$${\overset{―}{\gamma }}_{x2}=\frac{\left(1+\gamma_{x1})[\gamma_{x23}(1+\gamma_{x1})+\gamma_{x2}\eta_{y23}\right]}{\gamma_{x2}(1+\rho_{2}{)}^{2}}-\frac{\left(1+\gamma_{x1})[\gamma_{x13}+\gamma_{x2}\eta_{y13}\right]}{(1+\rho_{2}{)}^{2}}.$$

These coefficients govern the dynamics near the center manifold, incorporating the variables ${\overset{―}{x}}_{n}$, ${\overset{―}{y}}_{n}$, and $h^{*}$. Higher-order terms contribute to refining the approximation, providing a more accurate depiction of the system’s behavior in the neighborhood of the equilibrium.

${\overset{―}{x}}_{n+1}=-{\overset{―}{x}}_{n}+\sigma_{1}{\overset{―}{x}}_{n}^{2}+\sigma_{2}{\overset{―}{x}}_{n}h^{*}+\sigma_{3}{\overset{―}{x}}_{n}^{2}h^{*}+\sigma_{4}{\overset{―}{x}}_{n}h^{*2}+\sigma_{5}{\overset{―}{x}}_{n}^{3}+O((\left|{\overset{―}{x}}_{n}\right|+\left|h^{*}\right|{)}^{3})\equiv F({\overset{―}{x}}_{n},h^{*}),$ where


$${\overset{―}{x}}_{n+1}=-{\overset{―}{x}}_{n}+\sigma_{1}{\overset{―}{x}}_{n}^{2}+\sigma_{2}{\overset{―}{x}}_{n}h^{*}+\sigma_{3}{\overset{―}{x}}_{n}^{2}h^{*}+\sigma_{4}{\overset{―}{x}}_{n}h^{*2}+\sigma_{5}{\overset{―}{x}}_{n}^{3}+O((\left|{\overset{―}{x}}_{n}\right|+\left|h^{*}\right|{)}^{3})\equiv F({\overset{―}{x}}_{n},h^{*}),$$

For a period-doubling (PD) bifurcation to occur, the critical quantities $\xi_{1}$ and $\xi_{2}$ must both be nonzero, where:


$$\xi_{1}=\left(\frac{\partial^{2}F}{\partial \overset{―}{x}\partial h^{*}}+\frac{1}{2}\frac{\partial F}{\partial h^{*}}\frac{\partial^{2}F}{\partial {\overset{―}{x}}^{2}}\right){|}_{\left(0,0\right)},$$

and


$$\xi_{2}=\left(\frac{1}{6}\frac{\partial^{3}F}{\partial {\overset{―}{x}}^{3}}+{\left(\frac{1}{2}\frac{\partial^{2}F}{\partial {\overset{―}{x}}^{2}}\right)}^{2}\right){|}_{\left(0,0\right)}.$$

The following result summarizes the conditions and outcomes of this bifurcation:

**Theorem 2.** *The system undergoes a period-doubling (PD) bifurcation at the equilibrium point $\chi_{1}(x^{*},y^{*})$ for certain values of the parameter *h* within a restricted vicinity of ${\hat{\Theta }}_{\chi_{1}}$. This bifurcation takes place provided that $\xi_{1}\neq 0$ and $\xi_{2}\neq 0$. Moreover, the stability of the resulting period-two orbits depends on the sign of $\xi_{2}$: when $\xi_{2}>0$, the orbits are stable, while $\xi_{2}<0$ leads to unstable period-two orbits.*

## 4 Complex network

To investigate the behavior of the discrete prey-predator model described in (2) within the framework of complex networks, we analyze a network composed of *N* nodes, where the nodes interact via linear and diffusive couplings. Each node in the network functions as an independent two-dimensional dynamical system, governed by a set of discrete equations that mirror those in (2). Consequently, the state equations for the coupled network, capturing the dynamics of predator-prey interactions, can be formulated as follows.

$$x_{i}(k)=h_{1}(x_{i}(k),y_{i}(k))-e\sum_{j=1}^{N}G_{ij}h_{1}(x_{j}(k),y_{j}(k)),\;y_{i}(k)=h_{2}(x_{i}(k),y_{i}(k))-e\sum_{j=1}^{N}G_{ij}h_{2}(x_{j}(k),y_{j}(k)).$$ (13)

Here, *e* denotes the coupling strength, while *G* represents the normalized Laplacian matrix of the network. The normalized Laplacian matrix plays a crucial role in capturing the diffusive interactions among the nodes, ensuring that the coupling effects are consistently scaled across the network. The Laplacian matrix *G* is defined as:


$$G=I-D^{-\frac{1}{2}}\mathrm{\backslash Tilde}BD^{-\frac{1}{2}}.$$

In this context, *I* represents the N×N identity matrix, $\tilde{ℬ}$ denotes the adjacency matrix of the network consisting of *N* nodes, and *D* is the diagonal degree matrix whose diagonal elements are given by $D_{ii}=k_{i}$. Here, *k*_*i*_ corresponds to the degree of node *i*, defined as


$$k_{i}=\sum_{j=1}^{N}{\tilde{ℬ}}_{ij}=\sum_{j=1}^{N}{\tilde{ℬ}}_{ji}.$$

For example, consider a star network with 10 nodes. The adjacency matrix $\tilde{ℬ}$ for this network can be expressed as:


$$\tilde{ℬ}=[\begin{array}{cccccccccc} 0 & 1 & 1 & 1 & 1 & 1 & 1 & 1 & 1 & 1\\ 1 & 0 & 0 & 0 & 0 & 0 & 0 & 0 & 0 & 0\\ 1 & 0 & 0 & 0 & 0 & 0 & 0 & 0 & 0 & 0\\ 1 & 0 & 0 & 0 & 0 & 0 & 0 & 0 & 0 & 0\\ 1 & 0 & 0 & 0 & 0 & 0 & 0 & 0 & 0 & 0\\ 1 & 0 & 0 & 0 & 0 & 0 & 0 & 0 & 0 & 0\\ 1 & 0 & 0 & 0 & 0 & 0 & 0 & 0 & 0 & 0\\ 1 & 0 & 0 & 0 & 0 & 0 & 0 & 0 & 0 & 0\\ 1 & 0 & 0 & 0 & 0 & 0 & 0 & 0 & 0 & 0\\ 1 & 0 & 0 & 0 & 0 & 0 & 0 & 0 & 0 & 0 \end{array}].$$

This matrix represents a star network topology, characterized by a central node (1) that maintains direct connections with all other nodes in the network. In contrast, the peripheral nodes are exclusively linked to the central node.

The degree matrix *D* for the star network structure is defined as:


$$D=D_{ij}=\left\{ \begin{array}{cc} 9 & \text{if }i=1,j=1,\\ 1 & \text{if }i=j\text{ and }i\neq 1,\\ 0 & \text{if }i\neq j. \end{array}\right.$$

The system described in Eq (13) can be reformulated in the following matrix representation:


$$x(k+1)=(I-eG)h_{1}(x(k),y(k)),\;y(k+1)=(I-eG)h_{2}(x(k),y(k)),$$

where $x(k)=(x_{1}(k),x_{2}(k),\ldots ,x_{N}(k))$ and $y(k)=(y_{1}(k),y_{2}(k),\ldots ,y_{N}(k))$ represent the state variables of the system at time step *k*.

## 5 Numerical results and discussion

In this section, we validate the aforementioned theoretical results through numerical simulations, presenting the maximum Lyapunov exponents, phase portraits, and various bifurcation diagrams of the system (2) near the positive fixed point. To support our analytical findings, we conducted numerical simulations using hypothetical data. As our study is not grounded in empirical observations or theoretical predictions, the parameters for these simulations were chosen arbitrarily.

### 5.1 NS bifurcation simulation

Earlier, we investigated the role of *h* in initiating a Neimark–Sacker bifurcation. Now, we aim to examine the system’s progression as it transitions to more complex dynamics during this bifurcation. To achieve this, we will systematically vary individual parameters—such as *A*, or *h* itself—while keeping the others fixed. Through this approach, we will interpret the resulting dynamic changes within an ecological framework, uncovering emerging patterns and their significance for the system’s overall behavior.

For the model (2), we consider the parameter values


(r,k,A,p,B,a,β)=(1.37,0.77,1.1,0.21,0.68,0.18,0.6),

and initial conditions $\left(x_{0},y_{0})=(0.58,0.97\right)$, with the bifurcation parameter *h* varied over [5.5,6.2]. At the critical value *h* = 5.7318, the system undergoes a Neimark-Sacker (NS) bifurcation. The positive fixed point is ${\tilde{\chi }}_{1}=(0.579777,0.966295)$, and the Jacobian matrix at this point yields the characteristic polynomial $J(\rho )=\rho^{2}\;+\;1.44176\rho \;+\;1.0026$. The roots, $\rho_{1,2}=-0.720879\;\pm \;0.694935i$, have a modulus $|\rho_{1,2}|=1$ at *h* = 5.7318, and from (9) we get l=0.301569≠0, confirming the occurence of NS bifurcation.

**Fig 1:**
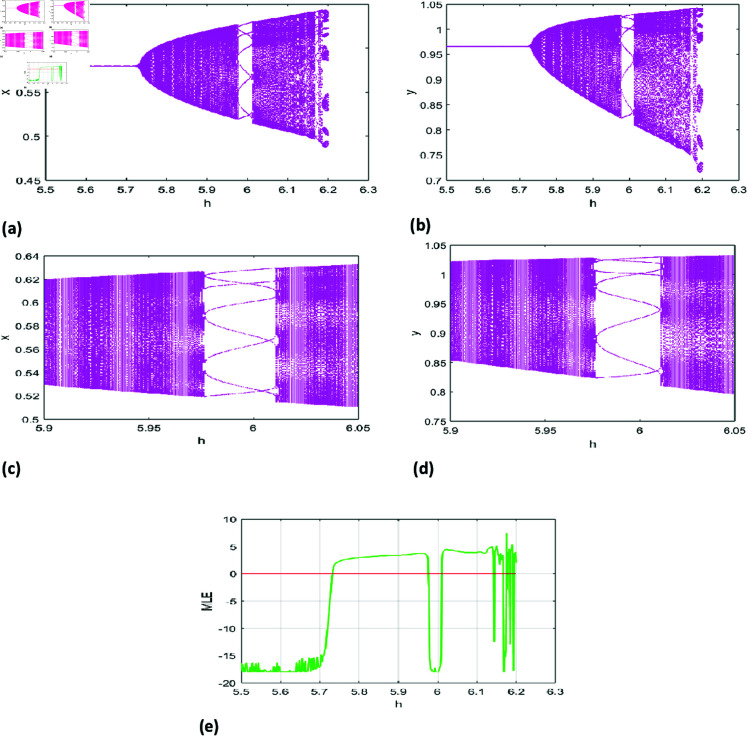
Bifurcation (NS) diagram of system (2) for r=1.37, k=0.77, A=1.1, p=0.21, B=0.68, a=0.18, β=0.6 initial conditions $({\text{x}}_{0},\;{\text{y}}_{0})\;=\;\;(0.58,\;0.97),$ and h lies in [5.5,6.2], (a) prey population, (b) predator population, (c,d) local amplification, (e) Maximum lyapunov exponent.

Fig 1(a), 1(b) illustrate the bifurcation diagram for h∈[5.5,6.2], while Fig 1(c), 1(d) provide a magnified view of local dynamics for h∈[5.9,6.05]. Fig 1(e) shows the maximum Lyapunov exponents, pinpointing the NS bifurcation at *h* = 5.7318.

The bifurcation diagrams (1) for *x* (prey population) and *y* (predator population) depict the system’s evolution as *h* varies. At low *h*, both populations stabilize at a fixed point, indicating equilibrium. As *h* increases, a Neimark-Sacker bifurcation occurs, destabilizing the fixed point and leading to quasi-periodic oscillations, visualized as a torus-like structure.

With further increases in *h*, the dynamics become increasingly complex, transitioning through secondary bifurcations to periodic or chaotic attractors. At high *h*, the diagrams reveal a dense scattering of points, characteristic of chaos, where the system exhibits sensitivity to initial conditions and unpredictability.

**Fig 2:**
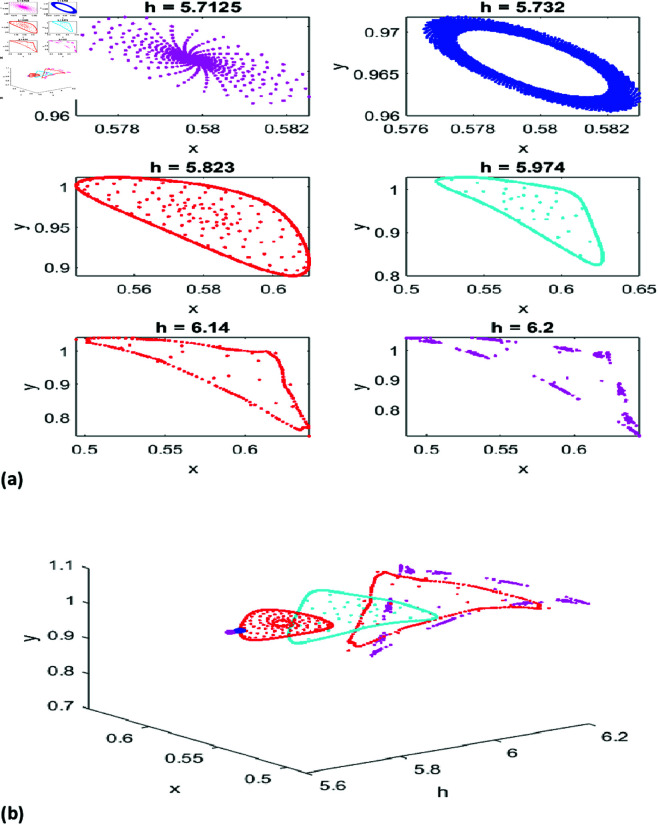
(a) Phase portrait for NS Bifurcation for different values of h. (b) 3D representation of phase portraits corresponding to Fig 2(a).

Fig 2(a) presents phase portraits for six values of *h*, illustrating the fixed point’s repelling nature. For *h* = 5.7125, 5.732, and 5.823, the phase portraits reveal diverse periodic behaviors. Fig 2(b) integrates these phase portraits with the *x*-*y*-*h* graph for a holistic perspective.

Together, the Neimark-Sacker bifurcation diagrams and phase portraits trace the system’s transition from stability to quasi-periodicity and chaos as *h* increases. These results highlight the sensitivity of predator-prey dynamics to parameter variations, underscoring the inherent challenges in predicting and managing complex systems governed by discrete dynamics. Such analyses are invaluable for understanding and mitigating periodic and quasi-periodic behaviors in ecological and economic contexts.

For the model [aa](2), we use parameter values


(r,k,h,p,B,a,β)=(1.37,0.77,5.73196,0.21,0.68,0.18,0.6),

and initial conditions $\left(x_{0},y_{0})=(0.58,0.97\right)$, with bifurcation parameter *A* varying over [0.9,1.28]. At *A* = 1.0990, the system undergoes a Neimark-Sacker (NS) bifurcation. The positive fixed point ${\tilde{\chi }}_{1}=(0.579777,0.966295)$ has a Jacobian matrix with characteristic polynomial $J(\rho )=\rho^{2}+1.44338\rho +1.00256$. The roots, $\rho_{1,2}=-0.721689\pm 0.694062i$, satisfy $|\rho_{1,2}|=1$, confirming the NS bifurcation.

**Fig 3:**
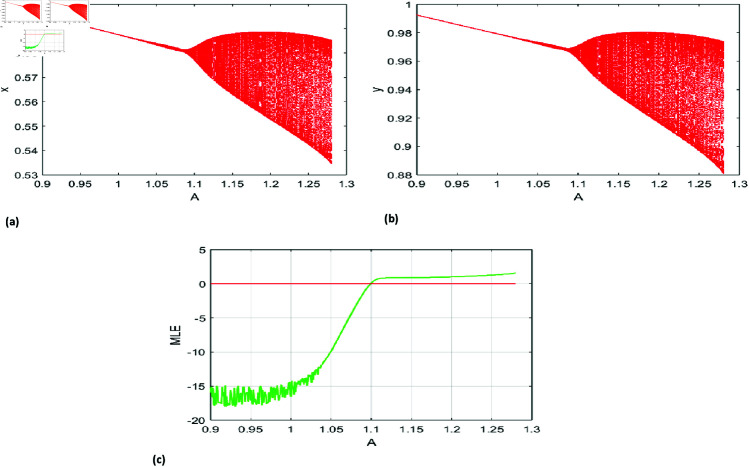
Bifurcation (NS) diagram of system (2) for r=1.37, k=0.77, h=5.73196, p=0.21, B=0.68, a=0.18, β=0.6 initial conditions $({\text{x}}_{0},\;{\text{y}}_{0})\;=\;(0.579777,\;0.966295),$ and A lies in [0.9,1.28], (a) prey population, (b) predator population, (c) Maximum lyapunov exponent.

Fig 3(a), 3(b) depict the bifurcation diagram for A∈[0.9,1.28], while Fig 3(c) highlights the maximum Lyapunov exponents, identifying the NS bifurcation at *A* = 1.1.

The bifurcation diagrams (3) for *x* and *y* depict the system’s behavior as *A* increases. A Neimark-Sacker bifurcation destabilizes the fixed point, giving rise to quasi-periodic oscillations and a torus-like structure. As *A* continues to grow, secondary bifurcations emerge, leading to periodic or chaotic attractors. At higher values of *A*, the diagrams display densely scattered points, characteristic of chaos and sensitivity to initial conditions.

**Fig 4:**
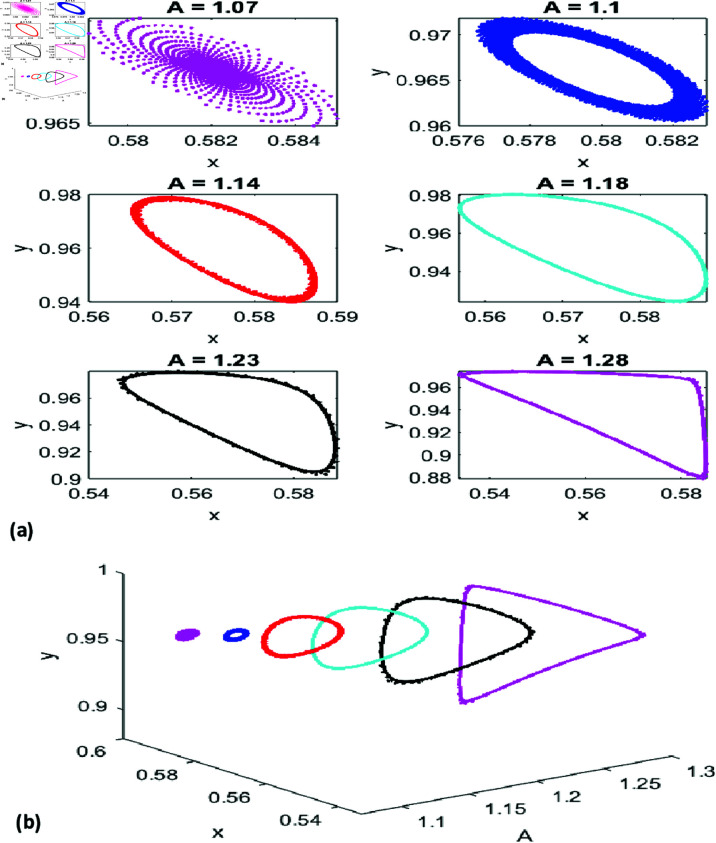
(a) Phase portrait for NS Bifurcation for different values of A. (b) 3D representation of phase portraits corresponding to Fig 4(a).

Fig 4(a) shows phase portraits for six values of *A*, highlighting the fixed point’s repelling nature and periodic behaviors for *A* = 1.1, 1.14, and 1.18. Fig 4(b) combines these with the *x*-*y*-*A* graph, illustrating the system’s transition from stability to quasi-periodicity and chaos as *A* increases.

**Fig 5:**
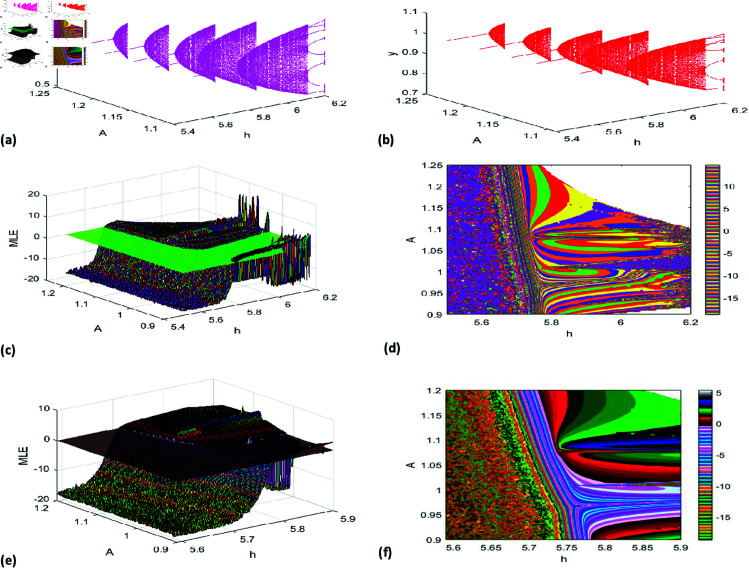
(a,b) 3D representation of Neimark-Sacker (NS) bifurcation diagrams for prey and predator populations (c–f) 2D & 3D MLEs.

The 3D bifurcation diagrams 5(a)-(b) illustrate the impact of *A* on system dynamics, showcasing transitions between stability, periodicity, and chaos. These visualizations provide a detailed perspective on the interplay between prey and predator populations and the conditions driving diverse regimes. Fig 5(c)–5(f) present 2D & 3D graph of the Maximum Lyapunov Exponent (MLE) for A∈[0.9,1.28] and h∈[5.5,6.2], highlighting multiple orbits in this range with close-up views of MLEs. Simulations align with the theoretical framework in Sect 3, revealing the complexity of NS bifurcations and the system’s sensitivity to parameter changes, offering key insights into ecological dynamics.

### 5.2 PD bifurcation simulation

The predator-prey model dynamics are defined using fixed parameters: *r* = 3.37, *k* = 0.77, *A* = 1.1, *p* = 0.21, *B* = 0.68, *a* = 0.18, β=0.6, and initial conditions $\left(x_{0},y_{0})=(0.69,1.14\right)$. The bifurcation parameter *h* varies within [1.55,1.8], with *h* = 1.6352 satisfying Proposition (2) and yielding the positive fixed point {x→0.685409,y→1.14235}. These values are used to generate Fig 7, showing prey and predator bifurcation diagrams alongside the MLE graph.

**Fig 6:**
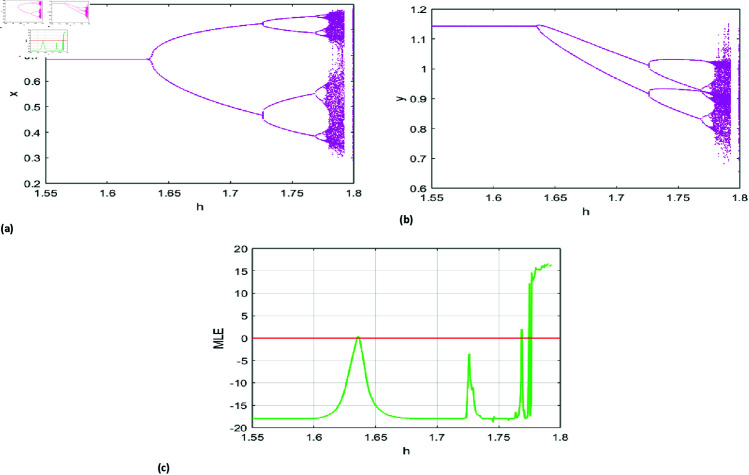
Bifurcation (PD) diagram of system (2) for r=3.37, k=0.77, A=1.1, p=0.21, B=0.68, a=0.18, β=0.6 initial conditions $({\text{x}}_{0},\;{\text{y}}_{0})\;=\;(0.69,1.14),$ and h lies in [1.55,1.8], (a) prey population, (b) predator population, (c) Maximum lyapunov exponent.

**Fig 7:**
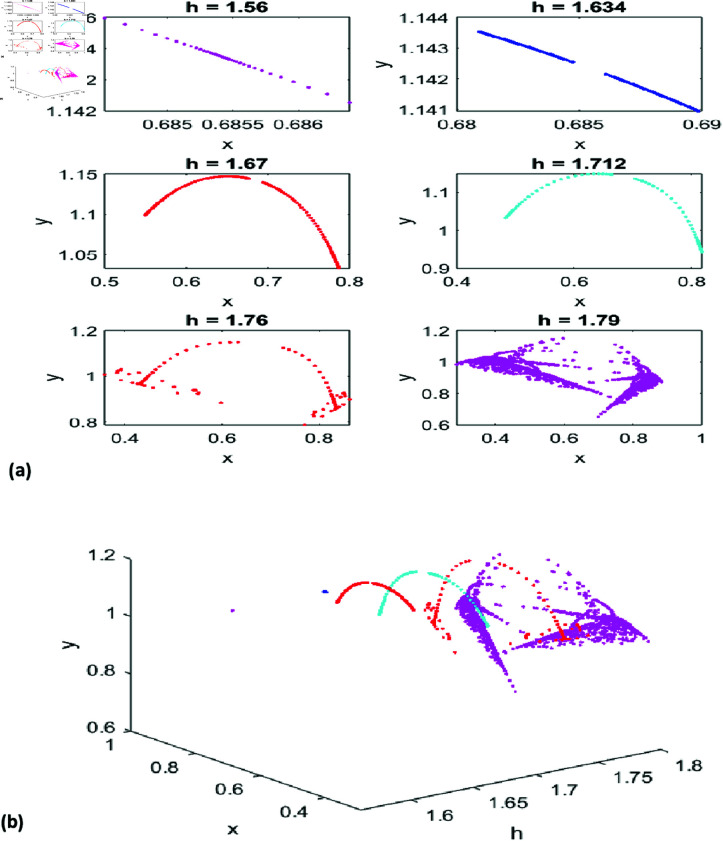
(a) Phase portrait for PD Bifurcation for different values of h. (b) 3D representation of phase portraits corresponding toFig 7(a).

**Fig 8:**
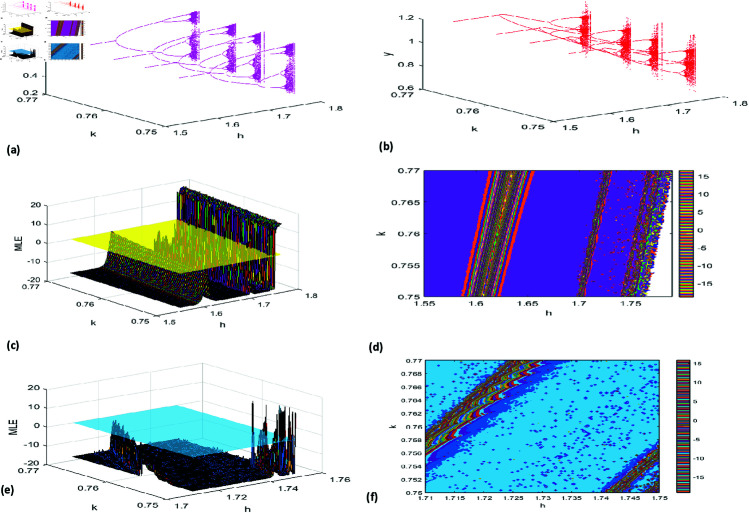
(a,b) 3D representation of Period-doubling (PD) bifurcation diagrams for prey and predator populations (c–f) 2D & 3D MLE.

Figs 6, 7, and 8 detail the bifurcation dynamics and stability of the predator-prey system under varying parameters. Fig 6(a) shows the prey population undergoing a period-doubling (PD) bifurcation as *h* increases from 1.55 to 1.8, reflecting significant behavioral changes. Similarly, Fig 6(b) depicts the predator population’s bifurcation with respect to *h*. The maximum Lyapunov exponent (MLE) graph in Fig 6(c) identifies stable and unstable regions, illustrating the system’s dynamic transitions.

Fig 7(a) displays phase portraits for six *h* values, showcasing oscillatory behavior, stability, and bifurcation patterns, while the 3D visualization in Fig 7(b) illustrates prey-predator dynamics across these *h* values. Fig 8(a), 8(b) extends the analysis with a 3D view of system dynamics as *k* varies from 0.75 to 0.77 and *h* spans its range. Additionally, Fig 8(c)–8(f) present the MLE with amplified detail, revealing how variations in *k* influence system stability and behavior.

The diagrams depict the evolution of a predator-prey system as the control parameter *h* increases. At low *h*, the populations reach a stable equilibrium, but as *h* rises, oscillations emerge, mimicking natural cycles. Further increases lead to period-doubling and complex dynamics, reflecting environmental impacts on population patterns. At high *h*, the system becomes chaotic, with unpredictable fluctuations, confirmed by the Maximum Lyapunov Exponent (MLE). These results highlight how small ecological changes can disrupt predator-prey stability, offering insights for ecosystem management and biodiversity conservation.

### 5.3 Simulation of complex network

**Fig 9:**
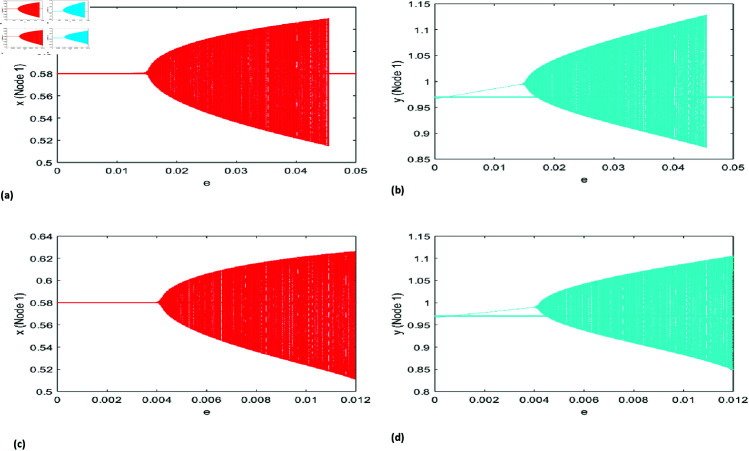
NS bifurcation diagram in the star network concerning the parameter e with r=1.37, k=0.77, A=1.1, p=0.21, B=0.68, a=0.18, β=0.6, h=5.6 initial conditions $({\text{x}}_{0},\;{\text{y}}_{0})\;=\;(0.58,\;0.97),$ (a) N=10 and e lies in 0\boldmath≤e\boldmath≤0.05 (b) N=50 and e lies in 0≤e≤0.012.

**Fig 10:**
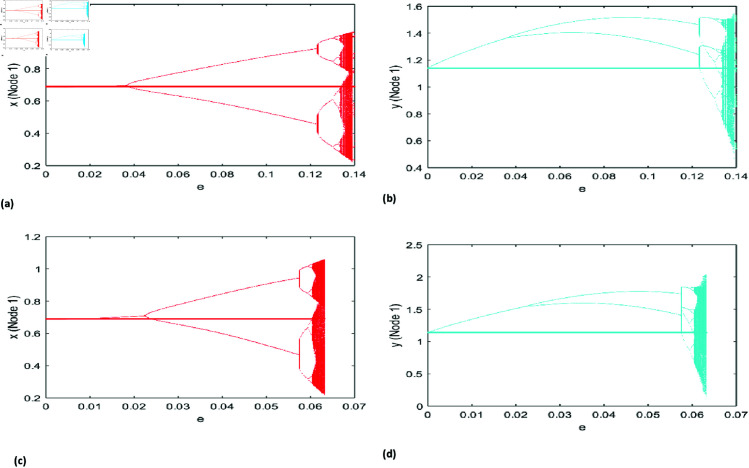
PD bifurcation diagram in the star network concerning the parameter e with r=3.37, k=0.77, A=1.1, p=0.21, B=0.68, a=0.18, β=0.6, h=1.6 initial conditions $({\text{x}}_{0},\;{\text{y}}_{0})\;=\;(0.69,\;1.14),$ (a) N=10 and e lies in 0≤e\boldmath≤0.14 (b) N=50 and e lies in 0≤e≤0.07.

The numerical simulation outcomes for the dynamics of our proposed model on star networks are shown in Figs 9 and 10. These results highlight the model’s behavior under different parameter configurations, emphasizing the impact of the star network’s structure on the system’s dynamics. The findings reveal key patterns and interactions influenced by the network topology, offering valuable insights into the modeled ecological processes. To demonstrate the occurrence of the NS bifurcation on star networks comprising *N* = 10 nodes, we set the coupling strength *e* = 0.0156678 and utilize the following model parameters as r=1.37,\breakk=0.77, A=1.1, p=0.21, B=0.68, a=0.18, β=0.6, h=5.6. For the star network consisting of *N* = 50 nodes, the parameter values remain identical to those used for the *N* = 10 node network. Fig 9(a) illustrates the NS bifurcation for the prey and predator populations in a star network with *N* = 10 nodes, while Fig 9(b) presents the corresponding bifurcation for a network with *N* = 50 nodes. The prey and predator population displays chaotic dynamics when the coupling parameter reaches a specific value, *e* = 0.004132. Notably, as the number of nodes increases, bifurcation and chaotic behavior arise at a lower coupling strength parameter value, as depicted in Fig 9(b).

Similar to the Neimark-Sacker (NS) bifurcation, our model demonstrates a period-doubling (PD) bifurcation. The dynamics of the PD bifurcation for a star network with *N* = 10 nodes are illustrated in Fig 10(a). The prey and predator populations experience their first PD bifurcation when the coupling parameter *e* exceeds the critical threshold of 0.03876. For the star network with *N* = 50 nodes, similar dynamics are observed; however, the bifurcation point shifts, and periodic windows of varying sizes emerge amidst chaotic behavior.

## 6 Chaos control

In the study of chaos control within discrete-time models, four primary strategies are frequently applied: the state feedback method, the OGY technique, the pole-placement method, and the hybrid control approach. Of these, the state feedback and OGY methods are the most widely utilized [26,27]. This section focuses on the OGY technique, which suppresses chaotic behavior by introducing small perturbations to system parameters. In contrast, the state feedback method employs real-time control inputs, derived from the system’s states, to regulate chaos with minimal intervention. The system (2) is expressed as follows:

$$x_{n+1}=x_{n}+h\left[rx_{n}\left(1-\frac{x_{n}}{k}\right)\left(\frac{1}{1+Ay_{n}}\right)-\frac{px_{n}y_{n}}{x_{n}+By_{n}}\right]\equiv f(x_{n},y_{n},\beta ),$$ (14)

$$y_{n+1}=y_{n}+h\left[ay_{n}\left(1-\frac{\beta y_{n}}{x_{n}}\right)\right]\equiv g(x_{n},y_{n},\beta ).$$

To regulate chaotic dynamics, we consider β as a control parameter and impose the condition $\left|\beta -\beta_{0}\right|<\tilde{h}$, where $\tilde{h}>0$, and $\beta_{0}$ represents a small value within the chaotic region. To achieve stability, a feedback control strategy is employed to guide the system’s solutions toward the desired target orbit. Assuming the system is at an unstable point, the approximate dynamics of the system, as defined in Eq (14), can be represented by the following mapping:

$$\left[\begin{array}{c} x_{n+1}-x^{*}\\ y_{n+1}-y^{*} \end{array}\right]\approx A\left[\begin{array}{c} x_{n}-x^{*}\\ y_{n}-y^{*} \end{array}\right]+B\left[\beta -\beta_{0}\right],$$ (15)

where


$$A_{\left(x^{*},y^{*},\beta \right)}=[\begin{array}{cc} \frac{\partial f(x^{*},y^{*},\beta )}{\partial x} & \frac{\partial f(x^{*},y^{*},\beta )}{\partial y}\\ \frac{\partial g(x^{*},y^{*},\beta )}{\partial x} & \frac{\partial g(x^{*},y^{*},\beta )}{\partial y} \end{array}]\;=\left[\begin{array}{cc} \frac{hr(k-2ℵ)}{Ak\hbar +k}-\frac{Bhp\hbar^{2}}{(B\hbar +ℵ{)}^{2}}+1 & hℵ\left(-\frac{Ar(k-ℵ)}{k(A\hbar +1{)}^{2}}-\frac{pℵ}{(B\hbar +ℵ{)}^{2}}\right)\\ \frac{a\beta h\hbar^{2}}{{ℵ}^{2}} & a\left(h-\frac{2\beta h\hbar }{ℵ}\right)+1 \end{array}\right].$$

Here, $ℵ=\frac{k(r(\beta +B)-p)}{Akp+\beta r(\beta +B)}$ and ℏ=βℵ. Moreover,


$$B_{\left(x^{*},y^{*},\beta \right)}=\left[\begin{array}{c} \frac{\partial f(x^{*},y^{*},\beta )}{\partial \beta }\\ \frac{\partial g(x^{*},y^{*},\beta )}{\partial \beta } \end{array}\right]=\left[\begin{array}{c} 0\\ -\frac{ah\hbar^{2}}{ℵ} \end{array}\right].$$

The controllability of system (14) is analyzed with respect to the matrix *C* given below:


$$\begin{array}{cc} C & =\left[B_{\left(x^{*},y^{*},\beta \right)}:A_{\left(x^{*},y^{*},\beta \right)}B_{\left(x^{*},y^{*},\beta \right)}\right]\\ & =\left[\begin{array}{cc} 0 & -\frac{ah\hbar^{2}}{ℵ}\\ -\frac{ah^{2}\hbar^{2}\left(-\frac{Arℵ\left(1-\frac{ℵ}{k}\right)}{(A\hbar +1{)}^{2}}-\frac{pℵ}{B\hbar +ℵ}+\frac{Bpℵ\hbar }{(B\hbar +ℵ{)}^{2}}\right)}{ℵ} & -\frac{ah\hbar^{2}\left(ah\left(1-\frac{\beta \hbar }{ℵ}\right)-\frac{a\beta h\hbar }{ℵ}+1\right)}{ℵ} \end{array}\right], \end{array}$$

The controllability matrix *C* is determined to have a rank of 2, confirming that the system is fully controllable. For the equilibrium solution of Eq (15) to maintain local asymptotic stability, all eigenvalues of the matrix A−BK must lie strictly within the open unit disk of the complex plane. If this condition is not satisfied, the solution becomes unstable and unsuitable for the intended analysis. Considering the feedback control law $\left[\beta -\beta_{0}\right]=-K[\begin{array}{c} x_{n}-x^{*}\\ y_{n}-y^{*} \end{array}],$ where $K=[\begin{array}{cc} d_{1} & d_{2} \end{array}],$ the system dynamics in Eq (15) can be reformulated to describe the closed-loop behavior as follows:

$$\left[\begin{array}{c} x_{n+1}-x^{*}\\ y_{n+1}-y^{*} \end{array}\right]\approx \left[A-BK\right]\left[\begin{array}{c} x_{n}-x^{*}\\ y_{n}-y^{*} \end{array}\right].$$ (16)

Here,

$$A-BK=\left[\begin{array}{cc} {ϝ}_{11} & {ϝ}_{12}\\ {ϝ}_{21} & {ϝ}_{22} \end{array}\right].$$ (17)

Consider,


$${ϝ}_{11}=h\left(\frac{r\left(1-\frac{ℵ}{k}\right)}{A\hbar +1}-\frac{rℵ}{k(A\hbar +1)}-\frac{p\hbar }{B\hbar +ℵ}+\frac{pℵ\hbar }{(B\hbar +ℵ{)}^{2}}\right)+1,$$

$${ϝ}_{12}=h\left(-\frac{Arℵ\left(1-\frac{ℵ}{k}\right)}{(A\hbar +1{)}^{2}}-\frac{pℵ}{B\hbar +ℵ}+\frac{Bpℵ\hbar }{(B\hbar +ℵ{)}^{2}}\right),$$

$${ϝ}_{21}=\frac{a\beta h\hbar^{2}}{{ℵ}^{2}}+\frac{ahd_{1}\hbar^{2}}{ℵ},$$

$${ϝ}_{22}=-\frac{a\beta h\hbar }{ℵ}+ah\left(1-\frac{\beta \hbar }{ℵ}\right)+\frac{ahd_{2}\hbar^{2}}{ℵ}+1.$$

The characteristic equation associated with the Jacobian matrix is expressed as:

$$\begin{array}{cc} P(\rho ) & =\rho^{2}-\Lambda \rho +\Upsilon =0. \end{array}$$ (18)

Let $\rho_{1}$ and $\rho_{2}$ denote the roots of the characteristic Eq (18). The parameters Λ and Υ are defined as follows:


$$\Lambda ={ϝ}_{11}+{ϝ}_{22},$$

$$\Upsilon ={ϝ}_{11}{ϝ}_{22}-{ϝ}_{12}{ϝ}_{21}.$$

The notations Λ and Υ represent the trace and determinant of the matrix A−BK, respectively. To define the boundaries of marginal stability, the conditions $\rho_{1}=\pm 1$ and $\rho_{1}\rho_{2}=1$ must hold. These criteria ensure that both eigenvalues, $\rho_{1}$ and $\rho_{2}$, remain strictly within the open unit disk in the complex plane, guaranteeing stability. Specifically, when $\rho_{1}\rho_{2}=1$, the corresponding values of Υ provide essential insights into the system’s stability characteristics and dynamic behavior.

The equations $\rho_{1}=\pm 1$ and $\rho_{1}\rho_{2}=1$ can be solved to determine the lines of marginal stability, which also ensure that both eigenvalues remain within the open unit disk. By consecutively considering the cases $\rho_{1}\rho_{2}=1$, $\rho_{1}=-1$, and $\rho_{1}=1$, the following equations are derived from (18):

$$L_{1}=\Upsilon -1,\;L_{2}=\Lambda -\Upsilon -1,\;L_{3}=1+\Lambda +\Upsilon .$$ (19)

Under certain parametric conditions, the stable eigenvalues are confined within a triangular region on the $d_{1}d_{2}$ plane, defined by the boundaries of the lines *L*_1_, *L*_2_, and *L*_3_.

**Illustration:** Consider the parameter values


$$(r,k,h,A,p,B,a,\beta_{0})=\left(1.37,0.77,5.8,1.1,0.21,0.68,0.18,0.595\right).$$

The system (2) achieves a coexistence fixed point at {x→0.578434,y→0.972158}. The modified system dynamics can then be described by the following equations:

$$x_{n+1}=5.8\left(\frac{1.37(1-1.2987x_{n})x_{n}}{1.1y_{n}+1}-\frac{0.21x_{n}y_{n}}{x_{n}+0.68y_{n}}\right)+x_{n},\;y_{n+1}=1.044y_{n}\left(1-\frac{0.595y_{n}}{x_{n}}\right)+y_{n}.$$ (20)

Let $K=\left[d_{1}\;d_{2}\right]$ represent the gain matrix, and let $\left(x^{*},y^{*})=(0.578434,0.972158\right)$ denote the equilibrium point of system (2), which inherently destabilizes the system. Furthermore, the system adheres to the following matrix relations:


$$A=(\begin{array}{cc} -1.43871 & -0.558981\\ 1.75462 & -0.044 \end{array}),$$

$$B=(\begin{array}{c} 0\\ -0.0775627 \end{array}),$$

and


$$C=(B:AB)=\left(\begin{array}{cc} 0 & -0.359312\\ -0.0775627 & -0.468828 \end{array}\right).$$

The rank of matrix *C* is 2, verifying that system (20) is fully controllable. Consequently, the Jacobian matrix of the controlled system (20), expressed as *A*−*BK*, is defined as:

$$A-BK=\left(\begin{array}{cc} -0.578434d_{1}-1.43871 & -0.578434d_{2}-0.558981\\ 1.75462\;-0.972158d_{1} & -0.972158d_{2}-0.044 \end{array}\right),$$ (21)

where A−BK represents the system’s modified dynamics matrix. The stability boundaries, defined by the lines *L*_1_, *L*_2_, and *L*_3_, are given by the following equations:


$$L_{1}=-0.0775627d_{1}-2.35517d_{2}-9.59997=0,$$

$$L_{2}=0.0775627d_{1}+2.71448d_{2}+2.3501=0,$$

and


$$L_{3}=-0.0775627d_{1}-1.99585d_{2}-12.8498=0.$$

**Fig 11:**
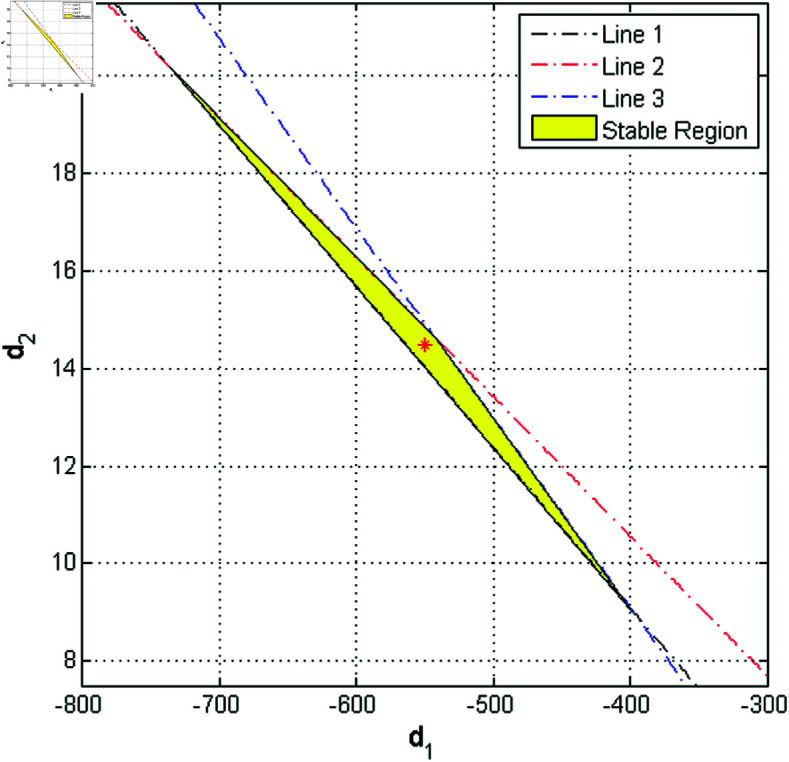
Stable eigenvalue region determined via the OGY control method.

The triangular region of stability, bounded by the lines *L*_1_, *L*_2_, and *L*_3_, is illustrated in Fig 11 for the controlled system described by Eq (20).

## 7 Conclusion

The fear effect describes the indirect impact, predators have on prey populations, where the mere presence or perceived threat of predators triggers changes in prey behavior, physiology, or spatial distribution, even without actual predation. Recognizing the ecological significance of fear is essential for conservation efforts, pest control strategies, and sustaining ecosystem equilibrium. Fear effects can contribute to long-term coexistence by encouraging prey to adopt adaptive strategies, such as spatial avoidance or altering their temporal activity patterns, which minimize direct interactions with predators. This study explores the intricate behavior of a discrete-time ratio-dependent Holling–Tanner predator-prey model that integrates the Fear effect. The research emphasizes the identification and stability of fixed points, alongside a detailed investigation of local bifurcations occurring at the positive fixed point. The study reveals that the system [aa](2) experiences both period-doubling and Neimark-Sacker bifurcations. Additionally, the system’s chaotic behavior is confirmed by detecting a positive maximum Lyapunov exponent (MLE). To mitigate bifurcations and chaos, the study applies the OGY control strategy. Theoretical findings are validated through numerical simulations using tools such as 2D and 3D bifurcation diagrams, MLE graphs, and phase portraits, providing a comprehensive visualization of the system’s dynamics. These results have significant biological implications, offering insights into the conditions leading to stable, oscillatory, or chaotic population dynamics in predator-prey systems. Understanding these dynamics is essential for developing effective strategies to manage and conserve ecosystems, promoting the sustainable coexistence of species in the long run.

Our results indicate that variations in the fear effect parameter induce bifurcations, positioning it as a key bifurcation parameter capable of stabilizing or destabilizing the predator–prey system. Biologically, a moderate level of fear appears to benefit both species, aligning with previous findings on the role of fear in shaping predator–prey dynamics. The stability of the positive fixed point reflects complex long-term behavior, highlighting the significant influence of fear on both prey and predator population dynamics.

Adapting the predator-prey model to a networked framework provides valuable insights into real-world ecological scenarios within interconnected systems. The results reveal that coupling strength and network structure influence the emergence of bifurcations and chaotic dynamics, emphasizing the importance of incorporating external interactions into predator-prey models. This study advances the understanding of nonlinear dynamics in predator-prey systems by employing modern mathematical methodologies and numerical simulations. Future research could explore the impact of asymmetric interactions and varied network structures on the behavior and stability of predator-prey dynamics.
